# Development of spontaneous and sensory evoked network activity in rodent cerebral cortex *in vivo*

**DOI:** 10.3389/fncel.2025.1648685

**Published:** 2025-08-06

**Authors:** Elena Nigi, Jenq-Wei Yang, Heiko J. Luhmann, Anne Sinning

**Affiliations:** Institute of Physiology, University Medical Center Mainz, Johannes Gutenberg University of Mainz, Mainz, Germany

**Keywords:** development, mouse, rat, neocortex, network activity, spontaneous activity, sensory evoked activity

## Abstract

Neuronal activity in the cerebral cortex comes in surprisingly early and influences or even controls a number of important developmental process like neurogenesis, neuronal migration, myelination, formation of cortical maps and local circuits, and programmed cell death. During the late prenatal and early postnatal period, the neocortical network shows a developmental transition from sparse, synchronized, low activity patterns to continuous, desynchronized, high activity patterns. This developmental sequence has been demonstrated in various neocortical areas of different mammalian species. This review article aims to provide a comprehensive overview of the early development of neuronal network activity in the cerebral cortex. We mainly focus on the rodent barrel cortex and a developmental period when the cortex gains mature functional properties at the cellular and network level. After briefly summarizing the developmental processes underlying the construction, reconstruction, and deconstruction of neocortical circuits, we describe the age-dependent changes in spontaneous and sensory driven network activity. Next we discuss the functional role of transient cortical structures and cell types in the generation of early activity patterns and in the activity-dependent maturation of local and large-scale cortical networks. Finally, we present an outlook on the models and techniques to study the cellular and network mechanisms underlying neuronal activity in the developing cerebral cortex.

## Introduction

Under unnatural *in vitro* conditions, in a dish with supply of artificial cerebrospinal fluid and nutrients, dissociated neurons from embryonic or neonatal mammalian brains have the surprising capability to generate spontaneous neuronal activity (action potentials) and to make synaptic connections to neighboring neurons. Within a few days *in vitro*, passive and active membrane properties (e.g., resting membrane potential and discharge pattern) reach more mature values similar to those obtained under *in vivo* conditions. With the functional maturation of inhibitory and excitatory synaptic connections, spontaneous (ongoing) activity becomes more complex and synchronized network activity emerges, recognizable as burst discharges, which can be local or propagate in a wave-like pattern across the cell culture network (Opitz et al., 2002; Sun et al., 2010). To some extent the development of intrinsic and synaptic activity recorded in various *in vitro* preparations, from dissociated cell cultures to brain organoids, resembles the developmental sequences described *in vivo* (for review Wu et al., 2024; Yuste et al., 2024). This is surprising since *in vitro* preparations (i) receive no sensory inputs, (ii) lack sensory-motor interactions, and (iii) are not influenced by neuromodulatory systems – factors that play important, if not essential roles in the physiological development of neuronal networks (for review Khazipov et al., 2008; Luhmann et al., 2016; Kilb and Fukuda, 2017; Martini et al., 2021; Cossart and Garel, 2022). In particular, they contribute as follows: (i) Already during perinatal development, the activity of thalamic or sensory cortical areas is driven by the sensory periphery, which plays a central role in the formation of cortical networks (Hanganu et al., 2006; Colonnese et al., 2010; Yang et al., 2013; Kersbergen et al., 2023; Aníbal-Martínez et al., 2025). (ii) Central pattern generators (CPGs) in spinal cord and brainstem produce spontaneous activity evoking motor commands and movements (Kreider and Blumberg, 2000; Khazipov et al., 2004), which subsequently activate sensory networks (An et al., 2014) and form early sensory-motor circuits. (iii) Neuromodulators have a fast and strong impact on cellular function and control the development of the neuronal network already at early stages (Crépel et al., 2007; Hanganu et al., 2007; Maldonado et al., 2021; Wong et al., 2022; for review Wu et al., 2024).

Distinct cortical activity patterns have been not only demonstrated during specific developmental stages in different mammalian species (for review Khazipov and Luhmann, 2006; Wu et al., 2024; Yuste et al., 2024), but also represent clinically important biomarkers of early physiological development in humans (Pavlidis et al., 2017; Routier et al., 2023; Kota et al., 2024). Cortical EEG patterns, spontaneous movements, and sensory-motor interactions provide important information on the functional state of the brain in newborns and can predict future clinical outcome (Iyer et al., 2015; Airaksinen et al., 2020; Lloyd et al., 2021). Therefore, a better understanding of the properties, underlying mechanisms, functions and dysfunctions of early network activity will form the basis for improvements in clinical diagnosis and therapies in neuropediatric (Luhmann et al., 2022).

The aim of this review article is to provide a comprehensive overview of the early development of spontaneous and sensory driven activity in the cerebral cortex. We focus on the developmental period when the cortical network gains mature functional properties at the cellular and network level, a process which in rodents mostly happens during the first postnatal month.

## Construction, reconstruction, and deconstruction of neocortical circuits during early development

During early development the cerebral cortex is characterized by highly dynamic changes in its molecular, anatomical and physiological properties. The initial cortical network consists of three layers: (i) The marginal zone, which beside other cell types, contains transiently expressed Cajal–Retzius neurons (CRNs) and later becomes cortical layer (L) 1 (for review Kirischuk et al., 2014; Causeret et al., 2021). (ii) The cortical plate, which is progressively populated by excitatory and inhibitory neurons. These neurons are generated in distinct brain regions and migrate along vertical and horizontal paths into the cortical plate to form the characteristic six-layered structure in an inside first – outside last sequence (for review Huttner, 2023). In the perinatal rodent cortex, one cortical layer is generated each day. (iii) The subplate, which contains a variety of transient neurons, and either disappears or later transforms into L6b (for review Hoerder-Suabedissen and Molnár, 2015; Kostović, 2020). During this dynamic postnatal phase of reorganization, CRNs, subplate cells, and a smaller number of neurons in L2–L6 are eventually eliminated by programmed cell death (apoptosis) (for review Causeret et al., 2018). These contrasting developmental processes – neurogenesis versus cell death and formation of new cortical layers versus loss of existing ones – are accompanied by the formation, stabilization and pruning of synaptic connections, developmental changes in synaptic function (e.g., number, affinity and subunit composition of receptors, location of synapses, and properties of transmitter release), and the increasing influence of neuromodulatory systems (e.g., cholinergic, serotonergic, and noradrenergic innervation). As a result, in rodents, the perinatal cerebral cortex profoundly changes its structure and function on a daily basis. This raises several important questions: How can the cortical network maintain its functional integrity under such dynamic conditions of construction, reconstruction and deconstruction? Is there functional stability in network activity, or do we see (abrupt) changes in network function during early development? Can we observe consistent patterns or gradual transitions of network activity during this dynamic period of development? What is the role of early activity patterns for the maturation of the cortical network? These questions will be addressed in the following subsections.

## Age-dependent changes in spontaneous network activity

It is the destiny of developing (cortical) neurons to generate spontaneous action potentials and to form synaptic connections. And it is the destiny of developing (cortical) networks to generate spontaneous synchronized burst discharges. These processes can be observed in simple cell cultures of dissociated neocortical neurons from newborn rodents (Opitz et al., 2002; Sun et al., 2010), in organotypic mouse neocortical slices (Bak et al., 2024), in human brain organoids (Trujillo et al., 2019; Landry et al., 2023), in rodent cerebral cortex *in vivo* (Minlebaev et al., 2009; Yang et al., 2009), and in EEG recordings from preterm human infants (Milh et al., 2007; Tolonen et al., 2007). While this list could be extended to other species and other brain structures, it remains unclear whether the network activity recorded under different conditions, in different models and in different species represents the same type of activity. Therefore, in the next sections we mostly focus on data obtained from the barrel cortex of rodents, which will be discussed in comparison to *in vitro* and *in vivo* observations from other neocortical areas and species.

Sparse spontaneous activity can be observed *in vivo* with calcium imaging and patch-clamp recordings in embryonic mouse neocortex as early as embryonic day (E) 14 (Yuryev et al., 2016; Munz et al., 2023). This is the period when genetic programs (e.g., spatio-temporal expression of specific transcription factors) and activity-dependent mechanisms interact (for review Simi and Studer, 2018). Between E14.5 and P2 spontaneous waves of activity can be monitored *ex vivo* with calcium imaging in sensory and higher-order thalamic nuclei of the mouse (Moreno-Juan et al., 2017). These calcium waves propagate via gap junctions across sensory-modality thalamic nuclei and regulate the formation of sensory neocortical areas (for review Antón-Bolaños et al., 2018). At E18, thalamic activity elicits a local, column-like activation of the subplate and the overlying cortical plate, suggesting that spontaneous thalamic activity instructs the columnar architecture and cortical maps before the arrival of sensory inputs (Antón-Bolaños et al., 2019; for review Martini et al., 2021). Interestingly, already during this developmental period, between E14.5 and E17.5, L5 pyramidal neurons form transient, multi-layered synaptic connections and participate in two different circuit motifs (Munz et al., 2023).

**FIGURE 1:**
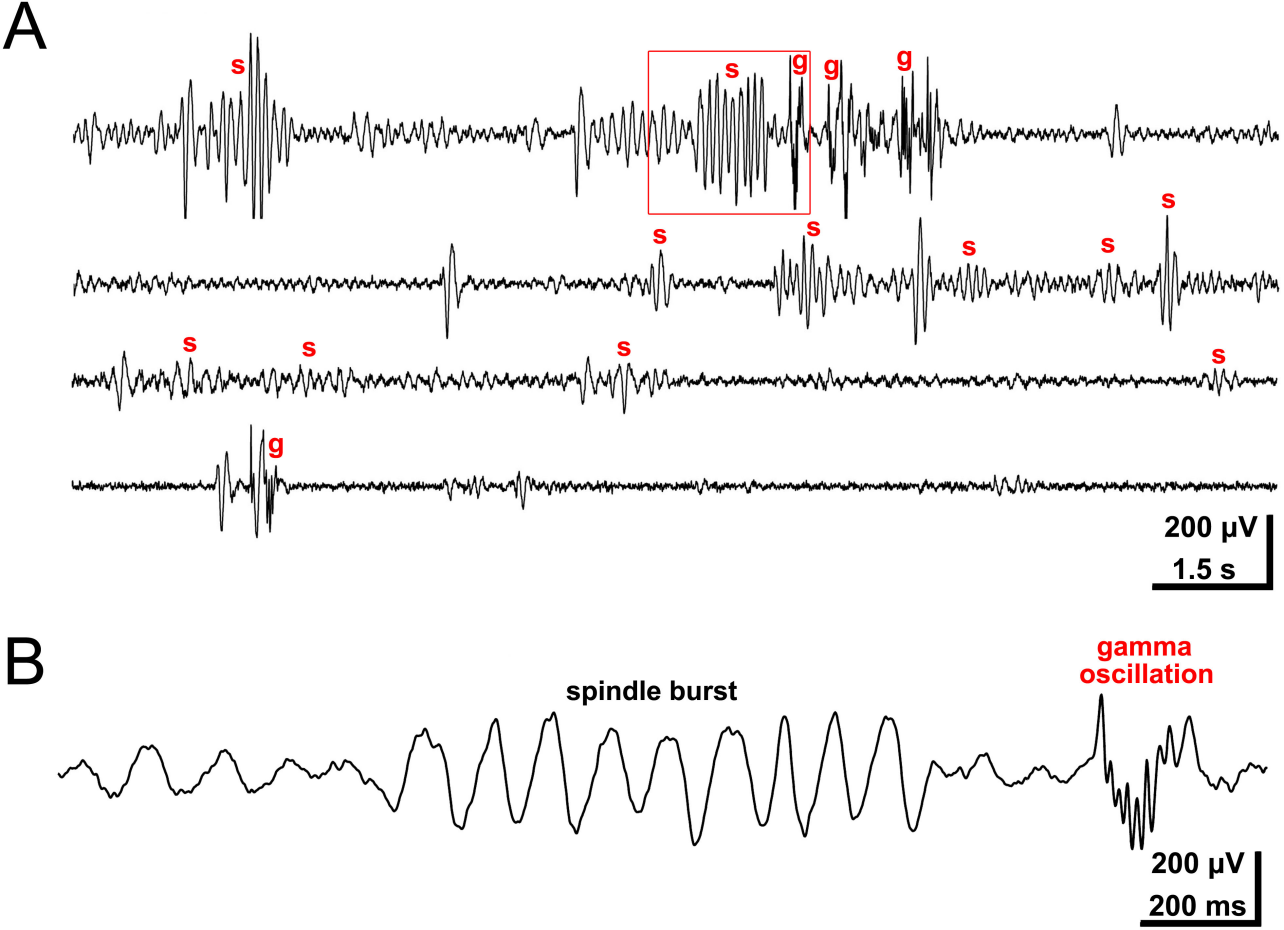
Intracortical recording of local field potential in barrel cortex of lightly anesthetized P3 rat. **(A)** Network activity consists of spindle bursts (s) and gamma oscillations (g). **(B)** Spindle burst and gamma oscillation marked in panel **(A)** by red rectangle at higher temporal resolution. Spontaneous and sensory-driven spindle bursts present with a dominant frequency in the alpha band (maximal frequency around 10 Hz), while intermittent gamma oscillations range around 30–40 Hz in frequency. Reproduced with permission from Yang et al. (2009).

**FIGURE 2:**
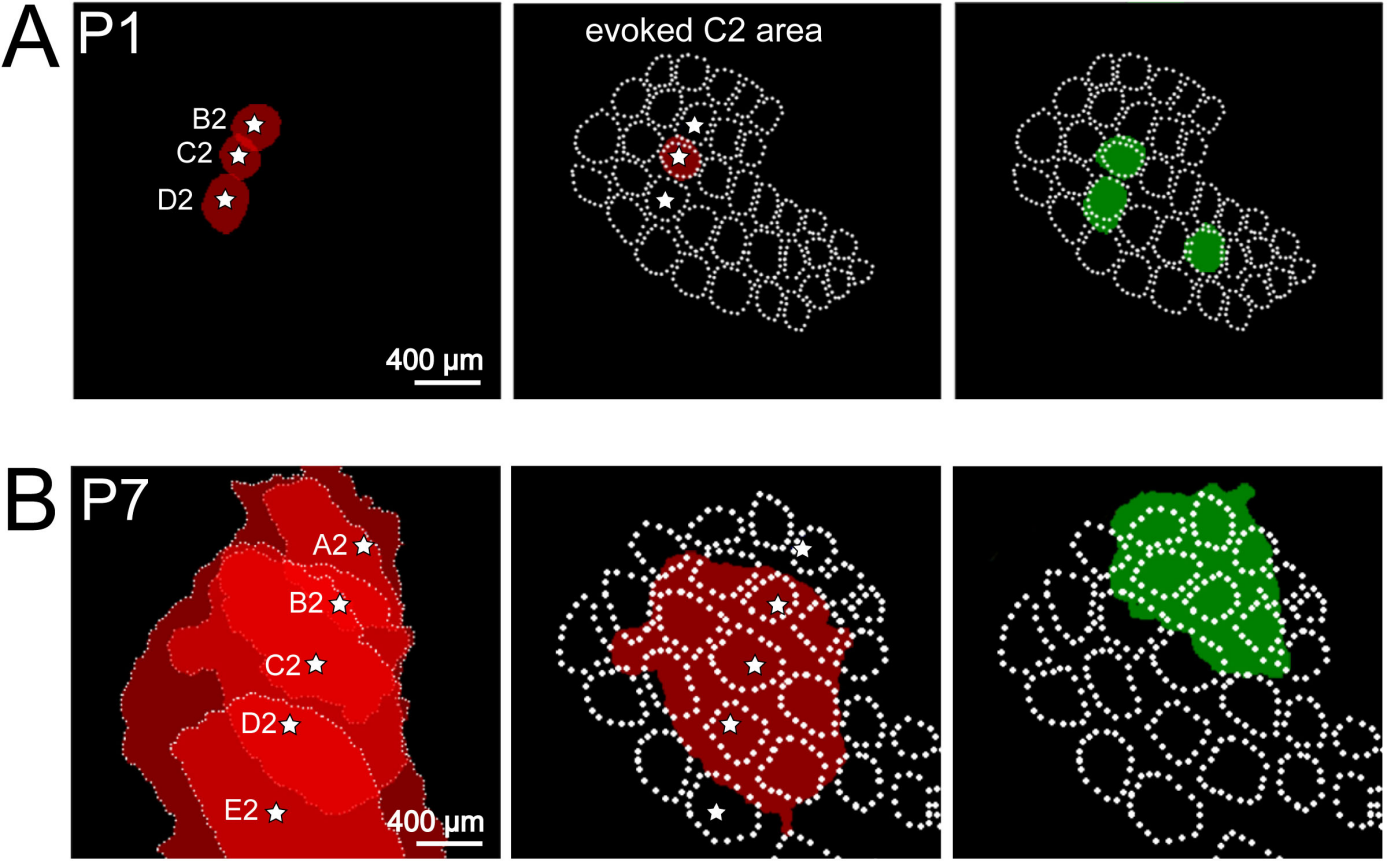
Spontaneous and evoked activity recorded with voltage-sensitive dye imaging (VSDI) in barrel cortex of P1 and P7 rat. **(A)** At P1, mechanical stimulation of single whisker B2, C2, or D2 elicits a local columnar response in the barrel field map (red circle-like areas). Local spontaneous events (green circle-like areas) overlap with the topographic representation of single whiskers indicating that spontaneous activity unmasks the functional barrel field map already shortly after birth. **(B)** At P7, single whisker (A2 to E2) stimulation elicits a wider response covering several columns. Spontaneous activity also covers several whisker-related columns. Reproduced with permission from Yang et al. (2013). White star marks the center of each activated barrel.

At P0, the day of birth, spontaneous neocortical activity patterns become more complex. Local, columnar domains of spontaneously coactive neurons as well as large-scale propagating activity can be observed *in vitro* and *in vivo* with calcium imaging, electrophysiological recordings and voltage-sensitive dye imaging (Yuste et al., 1995; Garaschuk et al., 2000; Minlebaev et al., 2007; Yang et al., 2009; Warm et al., 2025; for review Luhmann, 2017). Intracortical multi-electrode recordings *in vivo* have demonstrated that two distinct patterns dominate the spontaneous network activity in the neonatal rodent barrel cortex *in vivo*: local spindle-shaped oscillations (spindle bursts) and faster events in the gamma frequency range (gamma oscillations) (Minlebaev et al., 2007; Yang et al., 2009; Figure 1). Both patterns of spontaneous activity synchronize local, column-like networks (Figure 2A) and can be elicited by the thalamus (Minlebaev et al., 2011; Yang et al., 2013; for review Khazipov et al., 2013; Luhmann et al., 2016; Yang et al., 2016). In fact, in contrast to the activity in embryonic cortex, synchronized network activity in newborn rodent somatosensory, auditory and visual cortex can be triggered at this age by endogenous activity in the sensory periphery, e.g., retinal waves (for review Kirkby et al., 2013; Martini et al., 2021; Kersbergen and Bergles, 2024). A pivotal role in relaying these thalamic inputs is played by the subplate (Yang et al., 2009; Viswanathan et al., 2012), which is crucial in early circuit formation (for review Kanold and Luhmann, 2010; Luhmann et al., 2018; Molnár et al., 2020).

Beside activity from the sensory periphery activating the subplate-cortical plate network via thalamocortical connections, spindle bursts in newborn rat cortex are also modulated by interhemispheric connections via the corpus callosum (Marcano-Reik and Blumberg, 2008). These observations suggest that the subplate may serve as a transient hub station fulfilling important roles in cortical map and circuit formation. Selective removal of the subplate in newborn rat somatosensory cortex does not only abolish spindle burst activity, but disturbs the development of the characteristic barrel field pattern (Tolner et al., 2012). Thus, spontaneous burst activity transmitted via the subplate to the developing network in the cortical plate influences the formation of topographic maps and regulates intracortical connectivity (for review Kanold, 2009). Patch-clamp recordings in acute brain slices from postmortem human fetal cerebral cortex have demonstrated that during the second trimester of gestation subplate neurons spontaneously discharge in bursts and exhibit the highest level of functional differentiation when compared to other neocortical neurons (Moore et al., 2009; Moore et al., 2011). Gap junctions contribute to this spontaneous burst activity both in fetal human cortex (Moore et al., 2014) as well as in newborn rodent cortex (Hanganu et al., 2009; Singh et al., 2018). Conditional deletion of the gap junction protein Connexin 26 reduces spontaneous synchronized activity in newborn mouse neocortical slices and causes long-term behavioral deficits (Su et al., 2017), providing additional support that spontaneous burst activity at this developmental stage fulfills an important functional role.

With further development, spontaneous cortical activity becomes more complex, appears more often and frequently activates neighboring columns within the barrel cortex (Figure 2B) or propagates in a wave-like pattern across the cortex (Shen and Colonnese, 2016; Mojtahedi et al., 2021; Warm et al., 2025). At P4/5, when all neocortical layers have been formed, a patchwork-type of spontaneous activity pattern can be demonstrated by two-photon calcium imaging in L4 of the mouse barrel cortex (Golshani et al., 2009; Mizuno et al., 2018), indicating that the thalamus-driven columnar barrel field activation observed in P0/1 rodents (Yang et al., 2013) has shifted from the subplate to L4. During the first postnatal week, this transformation process in spontaneous activity patterns is accompanied by changes in intrinsic excitability (e.g., decrease in gap junctional coupling, age-dependent alterations in voltage-dependent currents) and an increasingly stronger participation of glutamate and GABA mediated synaptic transmission (Minlebaev et al., 2007, 2009; Mizuno et al., 2021; for review Moody and Bosma, 2005; Allene and Cossart, 2010; Luhmann et al., 2016; Yuste et al., 2024).

At the end of the second postnatal week (∼P14) spontaneous network activity in the mouse barrel cortex reveals a fundamental transition from high-frequency, synchronized activity to low-frequency, desynchronized activity (Golshani et al., 2009). A developmental desynchronization can be also observed in specific cell types, like the 5HT3a-receptor expressing interneurons, as demonstrated by longitudinal calcium imaging in barrel cortex of non-anesthetized mice (Che et al., 2018). Sequential maturation of GABAergic inhibition was recently shown to be critical for this network state transition in the barrel cortex (Modol et al., 2020; Mòdol et al., 2024). Further, a very similar development has been demonstrated in primary visual cortex of non-anesthetized rats (Colonnese et al., 2010) and mice (Shen and Colonnese, 2016). During the first postnatal week spontaneous activity is sparse with long (>10 s) periods of network silence. At the end of the second week, the time point of eye opening, network activity in visual cortex accelerates in frequency, becomes more continuous and resembles the adult pattern (Shen and Colonnese, 2016). Similar as in the barrel cortex, at this age spontaneous multi-unit activity (MUA) in the visual cortex increases, both in the awake state as well as during quiet sleep (Colonnese et al., 2010).

Moreover, spontaneous synchronized activity patterns in pre- and neonatal human cerebral cortex are remarkably similar to those observed in other mammalian species (for review Khazipov and Luhmann, 2006; Molnár et al., 2020; Chini and Hanganu-Opatz, 2021; Yuste et al., 2024). EEG recordings from preterm babies and *in utero* MEG recordings during the third trimester of pregnancy, demonstrated different physiological patterns of spontaneous activity (Vanhatalo et al., 2002; Rose and Eswaran, 2004; Vanhatalo and Kaila, 2006; for review Wallois et al., 2021). The spindle bursts described in rodents and other species resemble the so-called delta brushes recorded in preterm human babies (Milh et al., 2007; Kidokoro, 2021). As in other mammals, the cortical network activity in humans also shows a transition from sparse to discontinuous, synchronized and finally to continuous, desynchronized activity during late prenatal and early postnatal development (for review Wu et al., 2024).

Although the neocortical activity in this section was termed “spontaneous,” it has often not been clarified whether the spindle and gamma bursts are generated intrinsically within the cortical network or whether they arise from subcortical and non-cortical networks. Therefore, in the following section we focus on the role of the sensory periphery in triggering cortical network activity.

## Developmental changes in sensory driven cortical network activity

Spontaneous activity in sensory neocortical areas does not necessarily have to originate solely within intracortical circuits, but rather reflects the interaction of endogenous cortical activity with incoming thalamocortical, short- and long-range cortico-cortical inputs and the influence of neuromodulatory systems. In this section the role of the sensory periphery in triggering or modulating cortical network activity during early stages will be discussed.

The sensory periphery can trigger cortical network activity by two different mechanisms: through endogenous activity generated spontaneously within the sensory organs or by adequate physiological stimulation of the sensory cells. Both mechanisms occur surprisingly early – well before birth – in various sensory systems and across many mammalian species.

In the rodents’ visual system, spontaneous activity in the retina, called retinal waves, undergoes maturation in three developmental stages and plays an essential role for the refinement of visual maps in retinofugal targets in the thalamus and the superior colliculus (for review Blumberg et al., 2015). Retinal waves also trigger spindle bursts in the visual cortex of newborn rats *in vivo* (Hanganu et al., 2006). During the first postnatal week the visual cortex of non-anesthetized mice still cannot be activated by visual stimulation, but spindle bursts in response to retinal waves endure (Shen and Colonnese, 2016). Visual responses emerge as evoked spindle bursts at P8. In a laborious study Colonnese et al. (2010) investigated the development of spontaneous and evoked activity in the visual cortex of non-anesthetized rats (between P5 and P19) and preterm human infants (between gestational week GW27 and GW42). Their observations in the rat are almost identical to those obtained in the mouse. During the first postnatal week, sparse retinal wave-driven spontaneous spindle bursts could be observed in the visual cortex. Starting at P8, light flashes evoked visual cortical responses. *In vivo* patch-clamp recordings at this age demonstrated that the sensory evoked responses entail glutamatergic and GABAergic synaptic currents, which correlate with local field potential activity. Between P11 and P12 a rapid (<12 h) transition (“switch”) from light-evoked bursting to the more mature activity pattern could be observed. This developmental switch occurs shortly before eye opening at P13/14 and is accompanied by a sudden increase in mobility and explorative behavior (van der Bourg et al., 2017). A switch to the adult-like visual evoked activity pattern was also observed in EEG recordings from preterm human infants after GW36, which is shortly before term at GW37-40 (Colonnese et al., 2010). This transition from bursting to acuity is accompanied by the loss of gamma bursts organized in a columnar manner in rats and by the loss of delta brushes in preterms (Colonnese et al., 2010).

In the auditory system as well, the sensory periphery drives cortical activity during earliest developmental stages and spontaneous activity shapes the formation of the tonotopic map in primary auditory cortex (for review Kersbergen and Bergles, 2024). The mechanisms underlying spontaneous activity generation in the pre-hearing cochlea have been the focus of recent detailed studies: spontaneous calcium-dependent activity in glia-like supporting cells synchronizes the activity of nearby inner hair cells, which in turn increases the probability of afferent terminal recruitment (Wang et al., 2015; De Faveri et al., 2025). The network mechanisms causing sensory-driven activation of the auditory cortex during early developmental stages have been elegantly studied in ferrets, which are born at a very immature stage. Before the opening of the ears, sound-evoked activity in ferret auditory cortex emerges first in the subplate (Wess et al., 2017). At this age, abolishing peripheral function or presenting complex sound stimuli causes changes in the wiring pattern of subplate neurons (Meng et al., 2021; Mukherjee et al., 2022). Stimulus evoked responses can also be observed with standard EEG recordings in temporal cortical areas of preterm human infants (Chipaux et al., 2013), where the auditory responses consist of slow wave activity mixed with rapid oscillations. Thus, this delta-brush activity pattern is present in all cortical areas studied and resembles spindle bursts demonstrated in the perinatal cerebral cortex of other mammals (for review Khazipov and Luhmann, 2006).

**FIGURE 3:**
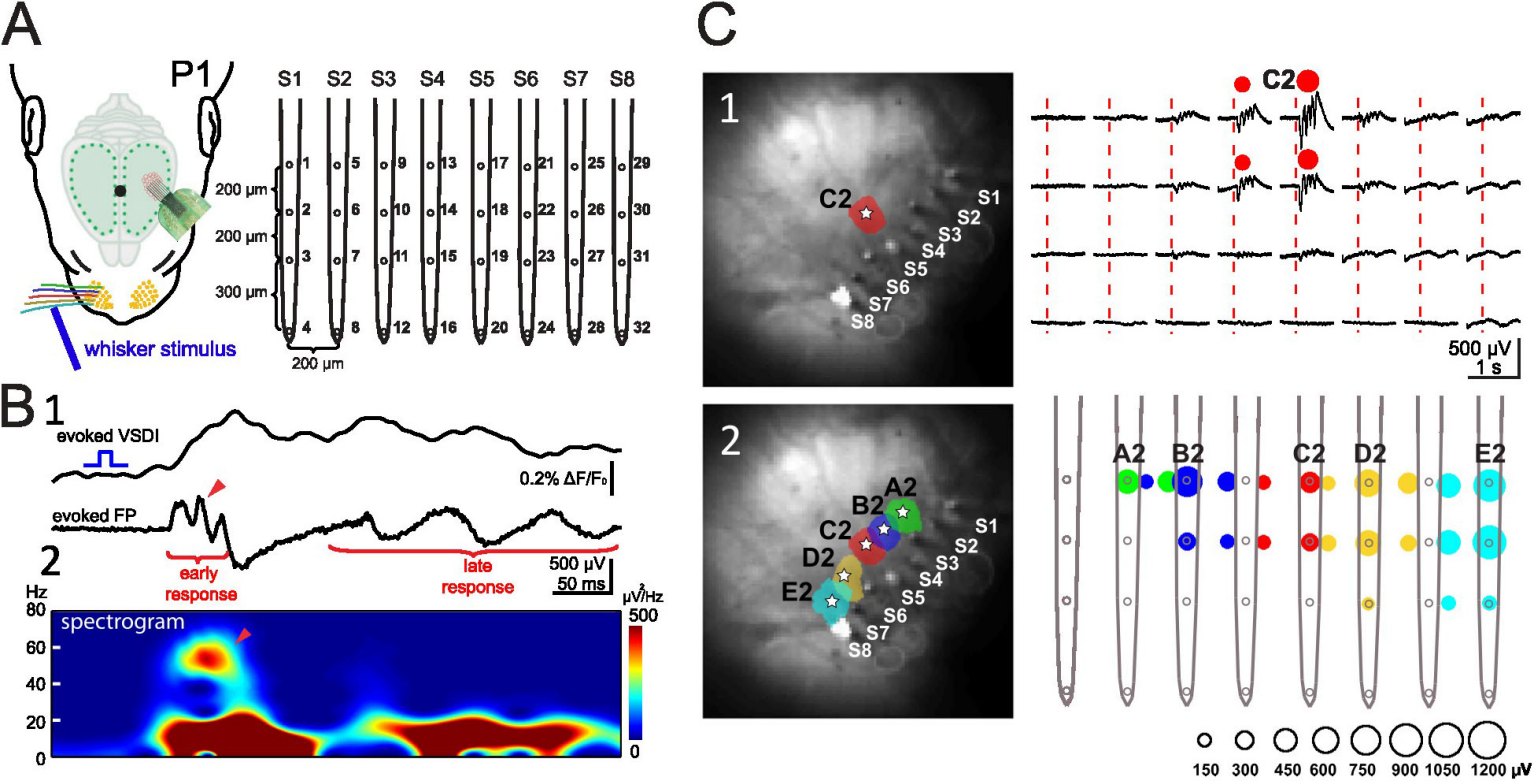
Evoked network activity recorded with VSDI and multi-electrode array in P1 rat. **(A)** Experimental setup showing the 8-shanks, 32-electrodes array. **(B)** VSDI and FP response recorded simultaneously in the cortical E2 barrel following mechanical stimulation of the E2 whisker. (B1) Simultaneous VSDI (upper trace) and local field potential (LFP) response (lower trace) to single whisker stimulation reveals early gamma oscillation and late spindle burst. (B2) Spectogram of the LFP shown in panel (B1) shows faster early gamma response followed by late spindle burst component. **(C)** Mechanical single whisker stimulation evokes a local columnar LFP response. (C1) Stimulation of whisker C2 elicits a local VSDI (left) and local electrophysiological (right) response in the C2 barrel. (C2) Color-coded localization of the evoked cortical VSDI (left) and electrophysiological (right) response amplitude to single whisker A2 to E2 stimulation. The LFP response amplitude corresponds to the size of the color-coded circles as shown below the graph. Reproduced with permission from Yang et al. (2013). White star marks the center of each activated barrel.

The role of the sensory periphery in triggering neuronal activity in the immature somatosensory cortex is particularly complex, as its diverse receptors (mechano-, proprio-, thermo-, and nociceptors) are distributed throughout the body and can activate various cortical networks. In this section we will mostly focus on the rodent barrel cortex, which is stimulated by the whiskers on the contralateral snout of the animal (for review Feldmeyer et al., 2013; Staiger and Petersen, 2021). Mechanical stimulation of a single whisker elicits a reliable response in the rat barrel cortex as early as P0/1 (Figure 3). Recordings with 8-shanks, 32-electrodes arrays covering a horizontal range of 1.4 mm and a depth of >700 μm allow the simultaneous recording of activity over several barrel-related columns and cortical layers in the barrel cortex of the neonatal rodent (Figure 3A). In the neonatal rat (Yang et al., 2013) the evoked whisker response consists of a short early component and a longer lasting late response. The early response shows the characteristic gamma oscillation frequency of 40–60 Hz and the late response oscillates in the spindle burst frequency of 10–20 Hz (Figure 3B). Both components are tightly coupled to the MUA (Yang et al., 2013). These intracortical multi-electrode recordings and voltage-sensitive dye imaging (VSDI) in barrel cortex of P0/1 rats (Yang et al., 2009; Colonnese et al., 2010; Yang et al., 2013) demonstrated single whisker stimulation elicited responses that were mostly restricted to a single cortical column (Figures 2A, 3C). Thus, sensory evoked responses as well as spontaneous activity in neonatal barrel cortex are organized in functional columns before structural columns can be firstly identified with anatomical and immunocytochemical methods at ∼P5 (for review Erzurumlu and Gaspar, 2012).

The question arises how and under which natural conditions the whiskers may be mechanically activated in a newborn rodent. Using a combination of multi-electrode recordings and video monitoring of the whiskers’ and head movements in non-anesthetized newborn rats, Akhmetshina et al. (2016) could demonstrate that whiskers move spontaneously and are also mechanically stimulated by tactile signals arising from its littermates’ movements. Both, endogenous self-generated whisker movements as well as exogenous stimulation by the littermates, efficiently evoke the typical early and late cortical response. What are the mechanisms underlying the spontaneous whisker movements? Rapid and asynchronous whisker movements have been demonstrated in P3–P6 rats during active sleep. These twitches are tightly coupled to bursts of activity in whisker thalamus and triggered cortical activity (Tiriac et al., 2012). However, this study does not fully explain the mechanisms of spontaneous whisker movements because a motor command has not been identified. Such a motor command may arise from the spinal cord, brainstem or motor cortex (for review Luhmann et al., 2016). In neonatal rats local spindle bursts in primary somatosensory cortex are triggered in a somatotopic manner by spontaneous muscle twitches (Khazipov et al., 2004) and the motor command for these twitches may arise from CPGs in the spinal cord. Sensory and motor zones of the spinal cord show synchronized activity, and twitches are correlated with movement-generating bursts in motor zones, which are followed by bursts in sensory zones (Inácio et al., 2016). The brainstem is another potential CPG for spontaneous motor activity during early development, since facial motor neurons in the brainstem elicit rhythmic whisker movements (Hattox et al., 2003). A whisking CPG may be also located in the primary motor cortex (M1). In awake adult rats, intracortical electrical stimulation of the M1 whisker representation elicits natural-like, rhythmic whisking (Haiss and Schwarz, 2005; Cramer and Keller, 2006). Notably, M1 is capable to trigger movements already in the neonatal rat, since focal electrical stimulation of the M1 forepaw representation in the physiologically relevant frequencies of spindle bursts (10 Hz) or gamma activity (40 Hz) reliably elicited movements of the contralateral forepaw (An et al., 2014). Approximately one quarter of the spontaneous bursts in M1 triggered forepaw movements and spindle bursts in M1 were tightly synchronized with spindle bursts in S1 (An et al., 2014). Carbocyanine dye (DiI) tracing experiments have shown reciprocal axonal connections between S1 and M1 in the P0 mouse and *in vitro* multi-electrode recordings demonstrated functional connectivity between these areas in the subplate and lower cortical layers at this age (Gellért et al., 2023). These data indicate that synchronized spontaneous activity in M1 and S1 mediated via corticocortical connections can influence maturation of sensorimotor interactions as early as P0 in rodents. The close interactions between the motor and the somatosensory system have been also nicely demonstrated with EEG recordings in preterm human infants. Prominent delta-brush activity can be observed in the somatosensory cortex, not only following a light touch of the baby’s shoulder, but also after spontaneous movements of the hand (Milh et al., 2007). Taken together this evidence suggests that these processes play a general role in the very early development of the sensorimotor system in mammals.

Using a combination of multi-electrode recordings, 2-photon calcium imaging and complex axial and/or lateral single whisker stimulation, van der Bourg et al. (2017) investigated evoked responses across all barrel cortical layers in anesthetized P10 to P28 mice. They found rapid functional changes at P13/14 marked by increased response selectivity and sharpened temporal spiking profiles. This developmental time point coincides with the onset of active whisking and exploratory behavior. In another study the same authors investigated in anesthetized P11 to P27 mice the responses to complex single- or dual-whisker stimulation (van der Bourg et al., 2019). Their results demonstrate a developmental decrease in the onset latency of the early response and duration of the late response, and an increase in the trans-columnar spread of the early activity. Herewith, these studies provide further evidence for a developmental sharpening of the temporal and spatial processing of whisker-evoked activity with the onset of active whisking.

The data discussed so far were mostly obtained in primary sensory cortical areas, such as the barrel cortex. Functional developmental changes in higher-order cortical areas have been less studied and remain largely unknown. Cai et al. (2022) used voltage-sensitive dye imaging, wide-field calcium imaging and intracortical multi-electrode recordings in anesthetized P0 to P56 mice to investigate the developmental engagement of the secondary whisker somatosensory area (wS2) and the emergence of cortico-cortical interactions from barrel cortex to wS2. They report that the thalamocortical input to wS2 is functionally delayed as compared to the thalamic input to the barrel cortex. Furthermore, cortico-cortical connections from barrel cortex begin to provide excitatory inputs to wS2 at P6–P8 and additional inhibitory inputs at P14–P16 (Cai et al., 2022). This developmental time point coincides with the sharpening of sensory evoked activity as exemplified by the shortening of evoked MUA. With the developmental transition of spontaneous activity from discontinuous and synchronized to continuous and desynchronized, sensory evoked activity interacts with an increasingly active cortex during network maturation. Thus, whereas sensory and thalamocortical activity encounter a relatively “silent” cortex during the first 10 postnatal days, patterned sensory inputs from the periphery reach a more “active” cortex in the succeeding stage. This developmental transition coincides with the period of eye opening, active hearing and voluntary movements (for review Martini et al., 2021), e.g., active exploratory whisking behavior (Arakawa and Erzurumlu, 2015).

## Role of early network activity in neocortical development

The developmental progression of functional properties in both spontaneous and stimulus evoked cortical activity patterns appears to be highly similar across the different sensory systems and all mammalian species studied to date (for review Wu et al., 2024). This suggests that early network activity plays an instrumental role in the development of the cerebral cortex during late prenatal and early postnatal stages.

Neuronal activity begins to influence cortical development at a surprisingly early stage. Spontaneous retinal waves at E15/16 control neurogenesis (cell cycle withdrawal), and pharmacological inhibition of retinal waves induces cortical layer malformations at later stages (Bonetti and Surace, 2010). Although the exact mechanisms underlying this activity-dependence and periphery-drive of neurogenesis are not yet clear, retinal waves may influence spontaneous calcium waves, which propagate through radial glial cells in the embryonic cortical ventricular zone (VZ) (Weissman et al., 2004; Yuryev et al., 2016). These waves are most prominent during the peak of neurogenesis in the VZ, and disrupting the calcium wave signaling pathway reduces VZ proliferation (Weissman et al., 2004).

The next step in corticogenesis, the migration of newborn cortical neurons to their final layer position, is also influenced by neuronal activity (Komuro and Rakic, 1996; De Marco García et al., 2011). NMDA receptors (Reiprich et al., 2005) as well as GABA-A receptors (Heck et al., 2007) are involved in this activity-dependent control of neuronal migration in the developing cerebral cortex. Subplate neurons participate in this process by forming transient NMDA receptor mediated synapses to multipolar neurons and inducing a change in the migration mode from slow multipolar migration to faster radial glial-guided locomotion (Ohtaka-Maruyama et al., 2018).

Recently it became clear that neuronal activity also influences the myelination process (for review Fields, 2015; Krämer-Albers and Werner, 2023). Release of glutamate from synaptic vesicles along active axons induces a local rise in cytoplasmic calcium in oligodendrocytes. This calcium signal stimulates local translation of myelin basic protein to initiate myelination (Wake et al., 2011; Wake et al., 2015). Furthermore, the activity-dependent rise in the extracellular potassium concentration also increases myelin basic protein synthesis (Friess et al., 2016; Hammann et al., 2018). Whether specific early activity patterns in the typical spindle and gamma burst frequency induce myelination in developing cortical axons is currently unknown.

During embryonic stages spontaneous activity influences the cortical patterning at the macroscopic scale (cortical arealization) followed by the patterning at the microscopic scale (cortical columns) (for review Martini et al., 2021). The impact of cell-intrinsic genetic programs (e.g., transcription factors) gradually diminishes during embryonic development (for review Simi and Studer, 2018). At late prenatal and early postnatal stages the subplate functions as an important relay and amplifier of thalamocortical inputs (for review Luhmann et al., 2018; Martini et al., 2018; Molnár et al., 2020). Subplate neurons are key elements in the generation of spindle bursts, and selective elimination of the subplate causes a disorganization of topographic cortical maps, such as the barrel field pattern (Tolner et al., 2012). In addition to subplate neurons, CRNs in the marginal zone/L1 are also activated by spontaneous thalamic inputs during prenatal stages. Thalamocortical activity regulates the density of CRNs, the distribution of upper layer interneurons and the dendritic activation of pyramidal neurons (Genescu et al., 2022). During the first postnatal week, reelin-positive interneurons in L1 of the barrel cortex, which co-express the serotonin 5-HT3a receptor, participate in a transient thalamocortical circuit and display spontaneous calcium transients synchronous with other superficial neurons (Che et al., 2018). Knockdown of NMDA receptors in these interneurons induces changes in whisker responses, barrel map formation and whisker-related behavior. Interestingly, this specific population of cortical interneurons also shows the typical developmental desynchronization from the first to the second postnatal week (Che et al., 2018).

**FIGURE 4:**
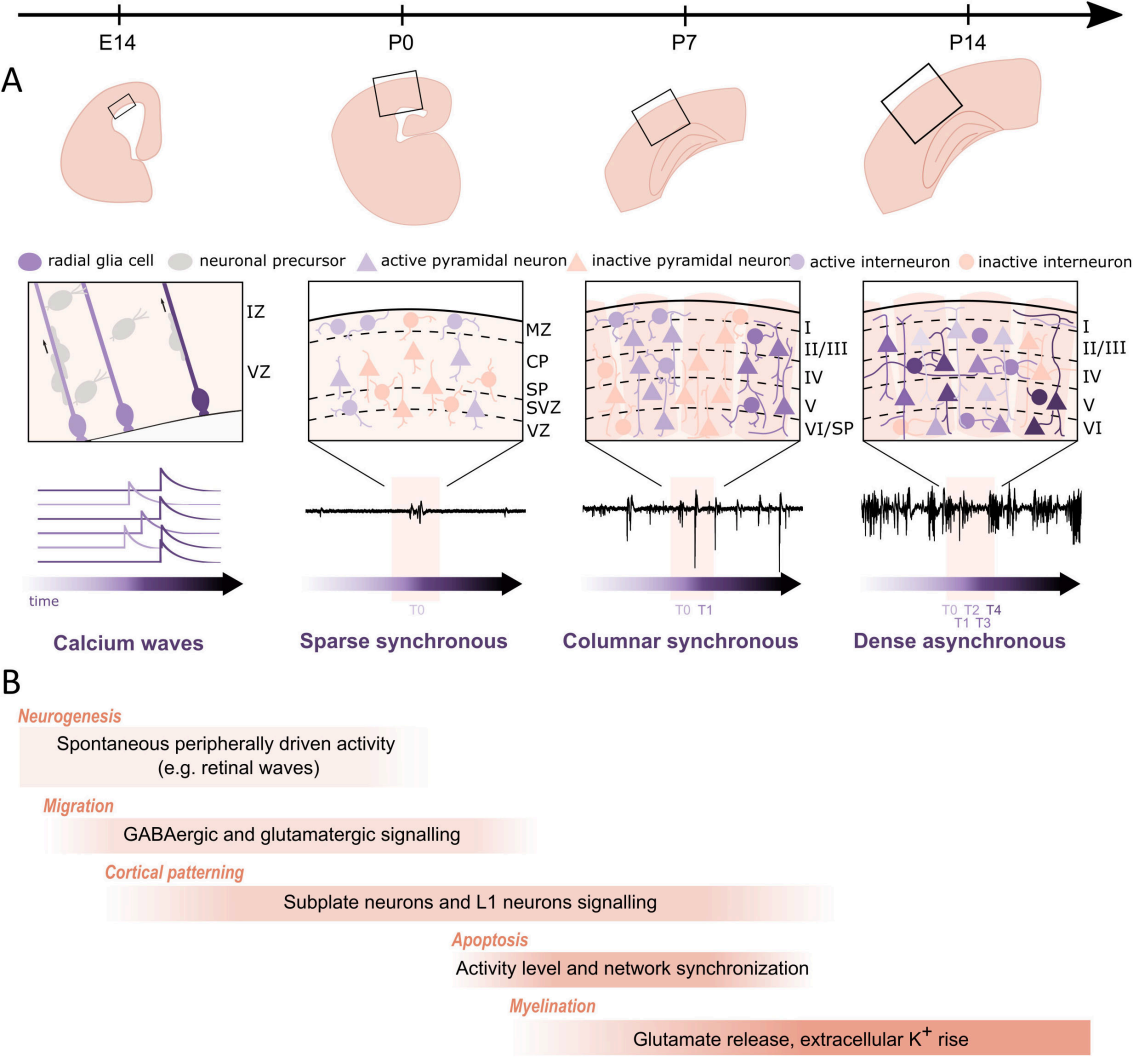
Regulation of cortical developmental processes by neuronal activity during late embryonic stage and the first two postnatal weeks in rodents. **(A)** Structural and functional maturation of the barrel cortex across the different perinatal stages. Top row: structural maturation at the macroscopic level. Middle row: close-up of the local circuitry. Bottom row: typical activity patterns (calcium signals at E14 and electrophysiological traces from P0 on) across the different developmental stages. At E14, slow calcium transients are observed in radial glial cells of the ventricular zone. At P0, sparse synchronous network activation underlies the sporadic and intermittent extracellular events. At P7, canonical synchronous columnar events drive more frequent bursts. At P14, the transition to dense and desynchronized activity generates the adult-like pattern. **(B)** Developmental processes that are regulated by spontaneous and/or evoked activity with illustrative mechanisms. During late embryonic stages, spontaneous calcium waves propagate through radial glia cells in the ventricular zone and promote proliferation. Next, activity drives radial and tangential migration of cortical neurons, e.g., through GABA-A receptor and NMDA-receptor mediated signaling. Subplate and layer 1 neurons serve as transient, intermediate relays and amplifiers of thalamocortical inputs and thereby facilitate the formation of cortical maps. During the first postnatal week a substantial subset of neurons is eliminated through apoptotic death, which is also regulated by intrinsic cellular and network activities. As neuronal activity increases, myelination of neurons is also triggered and remodeled, e.g., through synaptic glutamate release or an increase in extracellular potassium.

Beside neurogenesis, migration, myelination, circuit and cortical map formation, early neuronal activity also influences programmed cell death (apoptosis) (Figure 4). In the mouse cerebral cortex, 30%–40% of glutamatergic and GABAergic neurons are removed between P4 and P11 with a peak at ∼P7 (for review Blanquie et al., 2017; Causeret et al., 2018). The impact of spontaneous network activity on the E/I ratio by regulation of neuronal apoptosis has been elegantly studied with longitudinal calcium imaging in the somatosensory cortex of non-anesthetized mouse pups. During the first postnatal week, cortical interneurons deriving from the medial ganglionic eminence play a crucial role in the generation of GABA-driven neuronal dynamics underlying developmental apoptosis (Duan et al., 2020). The apoptosis process is regulated by specific activity patterns, which resemble the spindle burst activity observed during perinatal stage (Golbs et al., 2011; Wong Fong Sang et al., 2021). In a longitudinal *in vitro* study combining multi-electrode recordings and calcium imaging, Warm et al. (2022) could demonstrate that specific spontaneous activity features predict the survival or death of developing cortical neurons. High spontaneous firing rates, discharges in bursts, synchronized activity of close neighbors and large somatic calcium increases exerted a pro-survival effect. This activity pattern controls the downstream balance between mitochondrial pro- and anti-apoptotic factors, in particular BAX and BCL-2, which determines whether a cell survives or dies (Schroer et al., 2023). Further, the maturation of specific intracortical circuits is also regulated by activity-dependent apoptosis. GABAergic interneurons in L1 provide a major synaptic input to CRNs before the latter die (Kilb and Luhmann, 2001). The death of CRNs in the barrel cortex is crucial for the proper development of inhibitory projections from L1 interneurons to L2/3 pyramidal cells, the maturation of stimulus-evoked cortical responses and for the emergence of whisker-dependent behavior (Damilou et al., 2024).

It is not surprising that disturbances in the early activity patterns can have long-term consequences (for review Kirischuk et al., 2017; Hanganu-Opatz et al., 2021). In rodents, impaired gamma oscillations in the immature prefrontal cortex may induce neuronal and behavioral dysfunction resembling psychiatric disorders in humans (for review Chini and Hanganu-Opatz, 2021; Bitzenhofer, 2023). Disturbances in the activity patterns present in rodent cortex during the first postnatal week are typically characterized by aberrant neuronal synchrony and disrupted inhibitory interneuron activity, which is associated to neurodevelopmental disorders (for review Iannone and De Marco García, 2021). Early pathophysiological changes in spontaneous activity patterns may be of genetic origin. Perturbations of genes associated with schizophrenia and/or autism spectrum disorder (ASD) cause developmental disturbances in cellular and network function of the cerebral cortex. Tuberous sclerosis complex (TSC) is an autosomal dominant genetic disorder that induces brain tumors, intellectual decline, epileptic seizures, and ASD (for review Bassetti et al., 2021a). A mutation in TSC genes (Tsc1 and Tsc2) causes alterations in cortical E/I balance, which may be related to a dysfunction in glutamatergic or GABAergic synaptic function. Pyramidal neurons in the medial prefrontal cortex of Tsc2^+/^ mice reveal a weakening of tonic GABA-B receptor mediated tonic inhibition that increases neuronal intrinsic excitability and network excitability (Bassetti et al., 2020). Interestingly, the GABA-B agonist baclofen shifts the E/I ratio toward excitation only in Tsc2^+/^ neurons, but not in wildtype (Bassetti et al., 2021b). In another mouse model the early developmental switch between two distinct network states is disturbed by mutations in ASD associated genes (Munz et al., 2023). Non-genetic factors also influence spontaneous activity patterns in the developing cortex. Pro-inflammatory cytokines, including tumor necrosis factor (TNF) alpha, are released in response to maternal infection or perinatal hypoxia and induce a variety of neuronal and non-neuronal cascades (for review Deverman and Patterson, 2009). Experimental induction of inflammation by the application of the endotoxin lipopolysaccharide (LPS) to the newborn rat somatosensory cortex *in vivo* causes a rapid change in the pattern of spontaneous spindle bursts and gamma oscillations, which subsequently leads to an increase in apoptosis (Nimmervoll et al., 2013). This pathophysiological response to LPS-induced inflammation is mediated by a selective activation of microglial cells via the toll-like receptor-4 receptor. Activated microglia release TNF alpha and other pro-inflammatory factors, which modify the spontaneous activity patterns (Nimmervoll et al., 2013). Recently, Pöpplau et al. (2024) demonstrated with chronic extracellular recordings and optogenetic manipulations that microglia plays a central role in the developmental profile of early spontaneous network activity in mouse prefrontal cortex and that microglia ablation causes long-lasting disruption of cognitive prefrontal function.

In summary, synchronized neuronal network activity plays an important role in various important processes of early cortical development, from neurogenesis to formation of cortical maps and columnar modules. Disturbances of early activity by endogenous or exogenous noxae may have immediate, but also long-term consequences on cortical structure and function.

## Discussion

Using a wide repertoire of molecular biological, neuroanatomical, electrophysiological, and imaging techniques we gained a very good understanding of the early activity-dependent development of the rodent cerebral cortex. Comparative studies in other mammalian species demonstrate that the basic developmental sequences are very similar from mouse to human (for review Khazipov and Luhmann, 2006; Cossart and Garel, 2022; Yuste et al., 2024). We also learned that isolated *in vitro* models are rather limited in their relevance to comprehend activity-dependent developmental processes beyond the molecular and cellular scale. A lesson, which neurophysiologists have learned some decades ago (for review Steriade, 2001) and that already holds true at early embryonic stages, when development of the cerebral cortex depends on specific network input from subcortical regions and sensory periphery. Thus, an *in vitro* brain model without sensory organs, spinal cord and without a body, can only partly model the development of the brain (for review Nikolakopoulou et al., 2020; Luhmann, 2022).

The question arises, what would be currently the best model and technique to study the role of spontaneous and sensory evoked neuronal activity during early development. Obviously a whole animal model is required, preferentially in non-anesthetized recording conditions. Such recordings are technically less demanding in newborn rodents, which mostly sleep and exhibit only minor movements. However, the weight of the imaging or electrophysiological monitoring device is clearly a limiting factor, yet two-photon calcium imaging of the mouse embryonic cortex has been already successfully performed (Yuryev et al., 2016). Another requirement would be the monitoring of the same neurons over many days in the newborn mouse. Such a demanding experimental approach has been recently developed by Majnik et al. (2025) in the mouse barrel cortex. Their *Track2p* termed method allows the longitudinal cell tracking of several hundreds of individual neurons from daily two-photon calcium imaging over one postnatal week. With this powerful method the authors observed the sharp developmental transition from highly synchronized activity to multidimensional, behavior-state dependent neural dynamics centered on P11 (Majnik et al., 2025). Combining this experimental approach with acute or chronic optogenetic stimulation of genetically defined cell types, will provide further insights into the cellular mechanisms underlying spontaneous and evoked network activity in the developing cerebral cortex.
